# Increased Expression and Altered Methylation of *HERVWE1* in the Human Placentas of Smaller Fetuses from Monozygotic, Dichorionic, Discordant Twins

**DOI:** 10.1371/journal.pone.0033503

**Published:** 2012-03-21

**Authors:** Yu Gao, Zhiming He, Zilian Wang, Yanmin Luo, Hongyu Sun, Yi Zhou, Linhuan Huang, Manchao Li, Qun Fang, Shiwen Jiang

**Affiliations:** 1 Fetal Medicine Center, Department of Obstetrics and Gynecology, The First Affiliated Hospital of Sun Yat-Sen University, Guangzhou, Guang Dong Province, People's Republic of China; 2 Hoskins Center, Department of Biological Science, Mercer University School of Medicine, Savannah, Georgia, United States of America; 3 Department of Forensic Medicine, Sun Yat-Sen University, Guangzhou, Guang Dong Province, People's Republic of China; 4 Department of Obstetrics and Gynecology, Mayo College of Medicine, Rochester, Minnesota, United States of America; Université Paris-Diderot, France

## Abstract

**Background:**

The human endogenous retroviral family W, Env(C7), member 1 gene (*HERVWE1*) is thought to participate in trophoblast cell fusion, and its expression is diminished in the placentas of singleton intrauterine growth-retarded pregnancies. However, there is limited information about the role of *HERVWE1* in discordant fetal growth in twins. This study was to compare *HERVWE1* gene expression between the placentas of discordant monozygotic twins and to identify its regulation by methylation.

**Methodology/Principal Findings:**

Fetuses from twenty-one pairs of monozygotic, dichorionic, discordant twins were marked as “smaller” or “larger” according to birth weight. Placental *HERVWE1* mRNA and protein expression profiles were analyzed using quantitative RT-PCR and immunohistochemistry (IHC) staining. Methylation profiles of the *HERVWE1* promoter region were analyzed using a pyrosequencing assay. DNA methyltransferase (*DNMT*) transcript levels were analyzed by RT-PCR. 5-methyl cytosine (5-MC) was stained using an immunohistochemical assay. There was a significant negative correlation between *HERVWE1* mRNA levels and birth weight in twins (*P*<0.01). Whereas the mean methylation level of the *HERVWE1* promoter region was diminished in the smaller group in discordant twins(*P*<0.01), increased mRNA and protein levels of *HERVWE1* were found in smaller fetuses compared with larger fetuses in discordant twins(*P*<0.01). There was no significant difference in 5-MC staining intensity between discordant twins (*P*>0.05). The *DNMT3b3* mRNA levels in the smaller group were significantly downregulated compared with the larger group in discordant twins(*P*<0.05), whereas the *DNMT3b7* mRNA levels in the smaller group were significantly upregulated compared with the larger group in discordant twins(*P*<0.05).

**Conclusions/Significance:**

In discordant, monozygotic, dichorionic twins, *HERVWE1* expression was higher in smaller fetuses and lower in larger fetuses. Methylation of the *HERVWE1* gene promoter region may participate in the regulation of *HERVWE1* gene expression in discordant twin pregnancies.

## Introduction

The placenta plays an important role in fetal growth and development. Its key structure, a syncytium, maintains maternal-fetal nutrient transport and releases hormones. The structure of the syncytium in the placenta undergoes significant changes during intrauterine growth retardation (IUGR) [1]. Over the past few years, researchers have found that human endogenous retroviral family W, Env (C7), member 1 (*HERVWE1*), which is highly expressed in placental tissue, is the critical gene that regulates the action and preservation of the syncytium [1], [2]. The protein product of *HERVWE1* is syncytin-1, which mediates special functions in the placenta, including fusion, proliferation, anti-apoptosis, and immune suppression [3], [4]. The fusion function of syncytin-1 promotes the merging of cytotrophoblast cells to form the syncytiotrophoblast cell, which secretes several endocrine hormones that promote fetal growth, such as human chorionic gonadotrophin (HCG) and human chorionic somatomammotropin (HCS). *HERVWE1* expression is regulated by transcription factors, hormones, cytokines, environmental conditions, and DNA methylation. Chen and colleagues have proven that DNA methylation is one of the most important mechanisms for regulating *HERVWE1* [5]. Matouskova and Gimenez have shown that promoter region hypermethylation diminished *HERVWE1* transcript levels, whereas hypomethylation enhanced its transcript levels in placental and several non-placental tissues [6], [7]. Aside from the gene-specific promoter region methylation profile, global genomic methylation and the key enzymes regulating DNA methylation (DNA methyltransferases, DNMTs) may affect gene expression. In the last five years, *HERVWE1* expression has been found to be suppressed in IUGR placentas [8], [9]. However, previous research has focused on singleton pregnancies. The evidence in multiple pregnancies remains limited.

Monozygotic twins have identical inherited backgrounds and similar intrauterine circumstances. However, phenotypic discrepancies generally do exist between monozygotic twins [10], [11]. For example, birth weight discordance, which is defined as an inter-twin birth weight difference of greater than 20%, is one abnormal twin fetal growth pattern [12], [13]. An unbalanced placental blood supply caused by vessel anastomosis is one of the most important pathogeneses leading to weight discordance in monozygotic, monochorionic twins [14], [15]. However, this cannot explain the discordance in monozygotic, dichorionic twins as these twins share the same DNA sequence and similar circumstances, and there are seldom communicating branch vessels in the placenta. Unequal placental sharing has been identified in some discordant weight cases due to a lack of anastomoses [16]. However, the details of this molecular mechanism remain unclear.

Some researchers have studied the phenotypic differences between adult monozygotic twins and found that epigenetic modification is one of the most important causes [17]–[20]. However, most of these studies focused on adult diseases, such as psychiatric disorders [11], [21], [22], multiple sclerosis [20], [23], and systemic lupus erythematosus [24], and the study specimens were peripheral blood or epithelial cells. Few studies have examined intrauterine epigenetic modification in the placenta of discordant, monozygotic twins. Moreover, Fraga and coworkers pointed out that it takes a long time to accumulate significant changes in epigenetic modification [25]. It is still unclear whether epigenetic regulation affects fetal growth discordance during early life.

In this study, we investigated whether differential expression of *HERVWE1* and/or methylation of its promoter region were related to differences in the birth weight of twins, using gene expression and methylation analyses to examine placentas from monozygotic, dichorionic, discordant twins. In addition, we hypothesized that the key enzymes that regulate DNA methylation (DNMTs) contribute to the alterative methylation profile of the *HERVWE1* promoter region in monozygotic, dichorionic, discordant twins. As far as we are aware, this study is the first to use placenta samples from monozygotic, dichorionic twins to study *HERVWE1*. Our findings may increase our understanding of the role of the placenta in maintaining normal fetal growth in twin pregnancies.

## Material and Methods

### Case enrollment

All of the data for the twin pregnancies were collected at the First Affiliated Hospital of Sun Yat-Sen University, China, from July 2003 to December 2009. All intrauterine fetal deaths, IUGR, twin-twin transfusion syndromes (TTTS), and infants delivered before 26 gestational weeks were excluded from the study. Chorionicity was determined by a pathology exam after delivery. The total number of dichorionic twin pregnancies was 336. Among these cases, 134 pairs of different-sex twins were excluded due to dizygosity. Zygosity identification was applied in the cases of 202 pairs of same-sex twins. A total of 56 pairs were identified as monozygotic twins based on zygosity identification using a capillary electrophoresis assay. Among those pairs, there were 21 pairs of discordant twins for whom the birth weight difference was at least 20%. All 42 infants were enrolled into the larger or smaller group by pair according to birth weight. All of these cases were managed expectantly without any interventions. In addition, we collected data from 10 cases of singleton pregnancies delivered at 30–36 weeks for the singleton control group,and 24 pairs of concordant monozygotic dichorionic twins delivered at 30–36 weeks for the twins control group, for whom the birth weight difference was less than 20%. All the cases included in this study were delivered by cesarean section. All of the patients' parents or guardians were informed about the following research data collection. Consent was signed prior to enrollment. This study was approved by the Sun Yat-sen University Institutional Review Board.

### Sample collection

Within 15 minutes after delivery, placental tissue from around the individual insertion region of the umbilical cord was collected. The placental tissue was excised from inside the placental lobules, avoiding both the maternal surface and the amniotic membrane. Three 2×2 cm tissue samples were excised and washed 3 times in sterilized, ice-cold saline to eliminate any blood. One piece of tissue was placed in TRIzol solution for RNA isolation. The second piece of tissue was placed in a cryotube, which was then deep frozen in liquid nitrogen overnight and transferred to a −80°C freezer for storage prior to DNA isolation. The third piece of tissue was placed in a 10% formalin solution overnight and embedded in paraffin for future immunohistochemical staining.

### RNA/DNA extraction and conversion

RNA isolation was performed using TRIzol (Invitrogen, Cat. No. 15596-018, Carlsbad, CA, USA) and the phenol-chloroform method. The RNA was treated with Ambion DNA-free DNase treatment and removal reagents (Applied Biosystems, Part No. AM1906, Austin, TX, USA). The RNA was reverse transcribed to cDNA using a High Capacity RNA-to-cDNA kit (Applied Biosystems, Part No. 4387406, Austin, TX, USA). The cDNA was stored in a −20°C freezer until use in the real-time PCR assay.

DNA was extracted using the phenol-chloroform method. Bisulfite conversion was achieved with an EpiTect Bisulfite Kit (Qiagen, Cat No 59104, Valencia, CA, USA). Bisulfite-treated DNA was stored in a −20°C freezer until use in the pyrosequencing assay.

### Zygosity identification using a capillary electrophoresis assay

Genomic DNA was amplified with PowerPlex 16 (Promega, Cat No. DC 6531, Madison, WI, USA) in order to detect 15 autosomal short tandem repeat (STR) loci in addition to the gender determination marker amelogenin. Multiplex PCR was performed using 1 µl of template DNA in a 10 µl volume that included 1 µl of 10× GeneAmp PCR Gold Buffer, 1 µl of 10× Primer Pair Mix, and 0.3 µl of AmpliTaq Gold DNA polymerase (1.5 U) (Applied Biosystems, Cat No. 4338856, Carlsbad, CA, USA). The PCR cycling conditions were 95°C for 11 minutes; 96°C for 2 minutes; 10 cycles of 94°C for 1 minute, 60°C for 1 minute, and 70°C for 1.5 minutes; 22 cycles of 90°C for 1 minute, 60°C for 1 minute, and 70°C for 1.5 minutes; and a final extension at 60°C for 30 minutes. The PCR products were separated and detected by capillary electrophoresis using an ABI 3100 Genetic Analyzer (Applied Biosystem, Cat No. 4359571, Carlsbad, CA, USA) according to the manufacturer's instructions. Allele calls were made with GeneMapper ID v3.2 software (Applied Biosystem, Cat No. 4338856, Carlsbad, CA, USA). If all genotypes of the 16 loci were identical, then the pair was identified as monozygotic. If the genotypes of any loci were different, then the pair was identified as dizygotic. Appendix Figure S1 shows the 16 STR loci in one pair of monozygotic twins.

### Real-time PCR

To determine the transcriptional profile of *HERVWE1*, quantitative real-time PCR was used to compare *HERVWE1* mRNA levels between growth discordant twins, with three internal reference genes, glyceraldehyde-3-phosphate dehydrogenase (*GAPDH*),beta actin (*β-actin*), and proliferating cell nuclear antigen (*PCNA*).. The *HCS* gene located downstream of *HERVWE1* was identified as a factor reflecting placental function. The *HCS* mRNA level was measured as well. The concordant twins groups and singleton group served as normal controls. We also explored the transcription of three *DNMTs* (*DNMT1*, *DNMT3a*, and *DNMT3b*) and seven *DNMT3b* isoforms (*DNMT3b1-7*) in discordant twins and singleton control group using real-time PCR.

Briefly, cDNA was quantitatively analyzed by real-time PCR using a 7900HT fast real-time PCR system (Applied Biosystems, Part No. 4329001, Carlsbad, CA, USA). PCR was performed in 384-well plates using SYBR Green PCR Master Mix (Applied Biosystems, Part No. 4309155, Carlsbad, CA, USA). Each reaction contained 6 µl 2× SYBR green master mixture, 1 µl forward primer, 1 µl reverse primer, 1 µl cDNA template, and 3 µl H_2_O. Every reaction was repeated in quadruplicate. The thermal cycling conditions consisted of 50°C for 2 minutes; 95°C for 6 minutes; and 40 cycles of 95°C for 30 seconds, 45°C for 30 seconds, and 60°C for 1 minute. The quantitative gene expression results were analyzed with sequence detection system software (SDS v.2.3). The details of the real-time primers for *HERVWE1*, *HCS*, *DNMTs*, *GAPDH*, *β-actin*, *and PCNA* are shown in Appendix Table S1.

### Immunohistochemical staining (IHC)

Blocks from ten pairs of discordant twins, ten pairs of concordant twins, and ten singleton cases were available to apply the *HERVWE1* IHC staining. The same ten pairs of discordant twins and ten singleton cases applied the *5-MC* staining. The slices were stained under the same condition in *HERVWE1* IHC staining and *5-MC* staining respectively.

### HERVWE1 protein expression

Placental tissue was embedded in paraffin. Each slice was deparaffinized using xylene and gradient ethanol. Antigen retrieval was performed in boiling 10 mM citrate buffer for 10 minutes. The slices were then treated with 2 N hydrochloric acid (HCl) at 37°C for 30 minutes. Endo-peroxidase blocking was accomplished with 3% H_2_O_2_ in methanol for 20 minutes. Blocking of non-specific binding was performed with 1% gelatin. The samples were incubated at 4°C overnight with a 1∶100 dilution of affinity-purified rabbit polyclonal anti-human endogenous retrovirus IgG antibody (GeneTex Inc. Cat No. GTX70327, Irvine, CA, USA). A goat anti-rabbit secondary antibody conjugated to biotin (Vector Laboratories, Cat No. BA-1000, Burlingame, CA, USA) at a dilution of 1∶200 was applied for 30 minutes. The sections were then incubated with a Vectastain Elite ABC-peroxidase kit (Vector laboratories, Cat No. BA-1000, Burlingame, CA, USA) for 60 minutes. Staining was detected with a substrate solution of diaminobenzidine tetrahydrochloride (DAB) (Sigma Aldrich, Cat. No D5905-50, St. Louis, MO, USA). Counterstaining was performed with hematoxylin.

### Global methylation staining for 5-methyl cytosine (5-MC)

Tissue slices were incubated overnight in a 1∶200 dilution of sheep anti-5-Methyl Cytosine primary antibody (Capralogics, Inc., Cat No. P00704, Hardwick, MA, USA) followed by a 1∶200 dilution of biotinylated goat anti-sheep IgG (Vector Laboratories, Cat No. BA-6000, Burlingame, CA, USA) as the secondary antibody. The other steps were performed as described for the *HERVWE1* staining.

### Picture acquisition and processing

Images were captured using a Carl Zeiss microscope imaging system (Carl Zeiss, Cat. No. AxioVert 200 M, Thornwood, NY, US). Based on the proportion of positively stained cells and their degree of intensity, three random high-power fields (10×40) from each section were analyzed using AxioVs40LE digital image processing software (Carl Zeiss, AxioVision version 4.5.0.0, Thornwood, NY, US). The scoring system used to assess the trophoblast profile was as follows: negative (score = 0) = no positive staining, weakly positive (score = 1) = weakly positive staining seen within the structure, positive (score = 2) = positive staining seen within the structure, and strongly positive (score = 3) = strong staining signal within the trophoblast cell. The proportion of cells in each staining intensity category was multiplied by the trophoblast profile score to calculate the overall score.

### Pyrosequencing assay

The pyrosequencing assay was applied in all of the discordant twins and singleton control cases. There were 5 CG sites from −336 bp to −192 bp in the *HERVWE1* 5′LTR+U3 region. Appendix Figure S2 is a schematic diagram showing the CG sites in the *HERVWE1* promoter region. To analyze the methylation levels of the 5 CG sites, we used a pyrosequencing assay. The bisulfite-converted target DNA sequence was amplified using biotin-labeled primers, as shown in Appendix Table S1. Each PCR reaction contained 3 µl 10× PCR buffer, 1.8 µl 25 mM/L MgCl_2_, 0.6 µl 10 mM dNTP (Fermentas, Cat No. R0191, Glen Burnie, MD, USA), 0.15 µl Hotstar Taq DNA polymerase (Qiagen, Cat No. 203203, Valencia, CA, USA), 0.6 µl forward primer, 0.6 µl reverse primer, 1 µl DNA template, and 22.25 µl H_2_O. The thermal cycling conditions consisted of 95°C for 15 minutes; 45 cycles of 95°C for 30 seconds, 58°C for 30 seconds, and 72°C for 30 seconds; 72°C for 10 minutes; and 4°C until completion. The PCR products were sent to EpigenDx, Inc. (Worcester, MA, USA) for pyrosequencing. All new data had been deposited in GenBank. Because pyrosequencing can read only 50–100 bp for each accurate pyrosequencing primer, we used two pyrosequencing primers to analyze the five CpG sites. The pyrograms are shown in Appendix Figure S3. The methylation level of each CG site was calculated as the C/(C+T) peak height ratio. Unmethylated and in vitro methylated DNA were mixed at specified ratios to serve as controls.

### Statistical analysis

All of the data were analyzed using IBM SPSS v. 19.0 statistical software (SPSS Inc., Chicago, IL, USA). Clinical information was presented as the frequencies or means ± standard deviations. The comparisons between larger and smaller fetuses in twin pairs were performed using an independent sample t-test. The differences among the control groups, the smaller or larger discordant twin groups were analyzed using one-way ANOVA. The correlations between methylation levels and gene expression were evaluated using Spearman's correlation analysis. The semi-quantitative IHC data were analyzed using ANOVA with the least significant difference procedure (LSD) and post hoc multiple comparisons analysis. To remove the effect of gestational age, analysis of covariance was applied to evaluate the difference of gene expression in each group. A *P* value<0.05 was considered significant for all tests.

## Results

### Clinical characteristics

There were 21 pairs of monozygotic, dichorionic, discordant twins, 24 pairs of monozygotic, dichorionic, concordant twins, and 10 singleton pregnancies enrolled in our study. The clinical characteristics are summarized in Table 1. There were no differences in maternal age, race distribution, maternal body mass index (BMI), maternal parity, mode of delivery, delivery gestational age, incidence of maternal complication, or gender distribution among the discordant twins, concordant twins, and singleton groups. The birth weight of smaller fetuses in discordant group was the lightest among the five groups (*P*<0.01). The birth weight difference in discordant twins was significantly larger than that in concordant twins (*P*<0.05).

**Table 1:** Clinical characteristics among the groups.

	Larger twin in discordant group (n = 21)	Smaller twin in discordant group (n = 21)	Larger twin in concordant group (n = 24)	Smaller twin in concordant group (n = 24)	Singleton group (n = 10)
**Maternal age (years)**	27.95±4.22		28.38±3.98		27.60±2.07
**Race**					
**Asian (n, %)**	21 (100%)		24 (100%)		10 (100%)
**Non-Asian (n, %)**	0 (0%)		0 (0%)		0 (0%)
**Maternal BMI (kg/m^2^)**	24.6±3.8		24.2±3.6		22.1±3.2
**Maternal parity**	1.45±0.6		1.29±0.6		1.56±0.7
**Mode of delivery**					
**Vaginal delivery (n, %)**	0 (0%)		0 (0%)		0 (0%)
**Cesarean section (n, %)**	21 (100%)		24 (100%)		10 (100%)
**Delivery GA (weeks)**	32.87±2.57		33.69±2.08		33.41±2.43
**Sex distribution**					
**Male (n, %)**	16 (76.2%)		14 (58.3%)		6 (60%)
**Female (n, %)**	5 (23.8%)		10 (41.7%)		4 (40%)
**Maternal complications**					
**Preeclampsia (n, %)**	0 (0%)		0 (0%)		0 (0%)
**TTTS (n, %)**	0 (0%)		0 (0%)		_
**IUGR (n, %)**	0 (0%)		0 (0%)		0 (0%)
**GDM (n, %)**	0 (0%)		0 (0%)		0 (0%)
**Birth weight (kg)**	1.90±0.43*	1.37±0.36† ‡	2.06±0.37	1.88±.0.36	2.07±0.44
**Birth weight difference (%)**	28.5±5.3ξ		8.7±5.6		_

Data are shown as the means ± standard deviations or n (%).

BMI, body mass index; GA, gestational age; TTTS, twin-to-twin transfusion syndrome; IUGR, intrauterine growth restriction.

* P<0.01 vs. smaller twin in discordant group.

† P<0.01 vs. singleton group.

‡ P<0.01 vs. larger or smaller twins in concordant group.

ξ P<0.05 vs. concordant twin group.

### *HERVWE1* and *HCS* transcript levels

**Figure 1:**
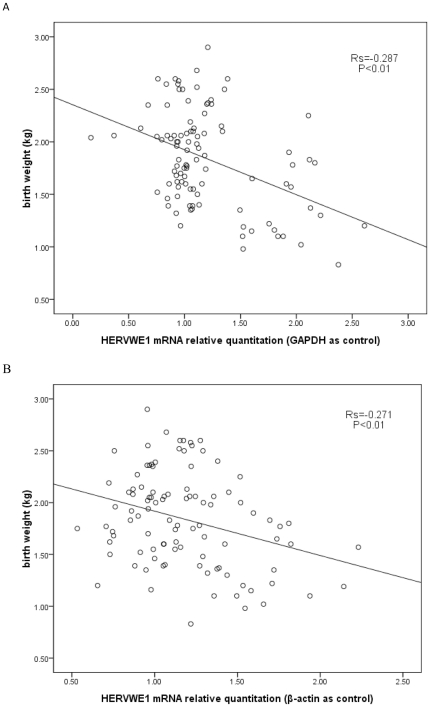
The correlation between *HERVWE1* transcript levels and birth weight. The scatterplots showed the correlation between the HERVWE1 transcript profile and the fetal birth weight in all five groups. (A) Using GAPDH as internal reference gene, the HERVWE1 transcript level was significant negatively correlated with the fetal birth weight (spearman correlation coefficient −0.287, P<0.01). (B) Using β-actin as internal reference gene, the HERVWE1 transcript level was significant negatively correlated with the fetal birth weight (spearman correlation coefficient −0.271, P<0.01).

In order to precisely investigate the *HERVWE1* transcript levels, three internal reference genes were applied. The *GAPDH* and *β-actin* were commonly used housekeeping genes. The *PCNA* was an index of cell proliferation which was used to eliminate the impact of cell proliferation on *HERVWE1*'s expression. As summarized in Table 2, the *HERVWE1* mRNA level of the smaller twin in discordant group was greater than that of the larger twin in discordant group (*P*<0.01, *GAPDH* and *β-actin* as control). The downstream gene *HCS*, as an indicator of placental function, also showed the same trend in discordant twins (*P*<0.05, *GAPDH* and *β-actin* as control). The *HERVWE1* and *HCS* transcript levels in the larger discordant twin group were similar to that in singleton group and two concordant twin groups (*P*>0.05). We observed an association between *HERVWE1* and *HCS* mRNA levels in the pool of all groups, with a Spearman's correlation coefficient (R_s_) of 0.647(*GAPDH* as control), 0.582 (*β-actin* as control) and 0.624 (*PCNA* as control)(*P*<0.01). We also observed a significant negative correlation between *HERVWE1* transcript levels and birth weight in all groups, with a correlation coefficient of −0.287 (*GAPDH* as control) and −0.271 (*β-actin* as control) (*P*<0.01) (Figure 1). We didn't find the correlation between *HERVWE1* transcript levels and delivery gestational age in each group (*P*>0.05). When gestational age was taken into account as covariate in covariance analysis, the same increased expression was detected in smaller fetuses in discordant twins.

**Table 2:** *HERVWE1* and *HCS* mRNA levels among the groups (*GAPDH*, *β-actin*, and *PCNA* as internal reference genes respectively).

	Larger twin in discordant group (n = 21)	Smaller twin in discordant group (n = 21)	Larger twin in concordant group (n = 24)	Smaller twin in concordant group (n = 24)	Singleton group (n = 10)
***HERVWE1***					
*GAPDH* as control	1.03±0.18	1.91±0.31†	0.96±0.22	1.04±0.29	1.00±0.09
*β-actin as control*	0.98±0.16	1.62±0.29†	1.09±0.22	1.13±0.26	1.00±0.17
*PCNA as control*	0.99±0.26	1.11±0.27	1.09±0.22	1.12±0.27	1.00±0.13
***HCS***					
*GAPDH* as control	1.12±0.33	2.17±0.36†	1.13±0.32	1.15±0.30	1.00±0.25
*β-actin as control*	1.18±0.29	1.73±0.35†	1.12±0.31	1.17±0.28	1.00±0.24
*PCNA as control*	0.97±0.34	1.07±0.28	1.08±0.31	1.09±0.25	1.00±0.24

The values shown indicate the relative quantitation of *HERVWE1* and *HCS* mRNAs, which were standardized to *GAPDH*,*β-actin*,*and PCNA* respectively, and normalized to the singleton control group.

The data are shown as the means ± standard deviations.

† P<0.01 vs. other four groups.

### *HERVWE1* protein expression

**Figure 2:**
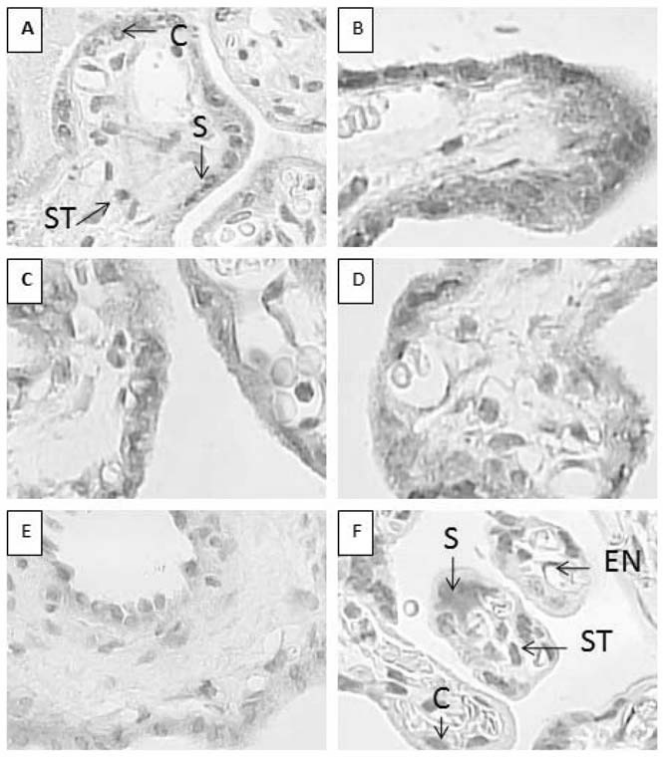
HERVWE1 staining of discordant twin, concordant twin, and singleton placentas. A–F show placental tissues with HERVWE1 immunohistochemical staining. The paraffin-embedded placental tissues were sliced into 4-mm sections. The slices were incubated in a rabbit polyclonal anti-human endogenous retrovirus IgG primary antibody (diluted 1∶100). A goat anti-rabbit secondary antibody (diluted in 1∶200) was applied sequentially. The slices were developed with an immunoperoxidase system. The HERVWE1 antigen was mainly present in the trophoblast cytoplasm. The positive cells were stained brown, and the negative cells were stained blue after counterstaining with hematoxylin. (A) The larger infant in discordant group (magnification 10×40). (B) The smaller infant in discordant group (magnification 10×40). (C) The larger infant in concordant group (magnification 10×40). (D) The smaller infant in concordant group (magnification 10×40). (E) A singleton infant (magnification 10×40). (F) The negative control (magnification 10×40). This singleton tissue slice was stained with 1% gelatin instead of the HERVWE1 antibody. Syncytiotrophoblast, cytotrophoblast, stromal cells, and endothelial cell are marked as S, C, ST, and EN respectively.

*HERVWE1* IHC staining images are shown in Figure 2. There was no significant difference in pathological structure examination among groups. All of the villous trees were misaligned and crowded, and the stem villi were scarred. The stem villi were short and thick. The majority of the villi were terminal villi, and they were tiny and irregular. The terminal villus was covered with a trophoblastic surface, which was composed of two layers. The villous syncytiotrophoblast constituted the outer continuous thin layer. The villous cytotrophoblast constituted the inner discontinuous layer. Inside the villi was the stroma, including the fetal vessels, macrophages, and connective tissue fibers. The positive staining of *HERVWE1* was mostly concentrated in the cytoplasm of the trophoblast cells. There was no significant difference in syncytiotrophoblast or cytotrophoblast cell number among all groups.

We compared the intensity of trophoblast cells among the discordant twin group, concordant twin group, and singleton group (Table 3). The smaller twin in discordant group had the strongest *HERVWE1* protein expression among the five groups (*P*<0.01). There was no significant difference among the larger twin in discordant group, two concordant twin groups, and the singleton group (*P*>0.05).

**Table 3:** *HERVWE1* staining scores among the groups.

	Larger twin in discordant group (n = 10)	Smaller twin in discordant group (n = 10)	Larger twin in concordant group (n = 10)	Smaller twin in concordant group (n = 10)	Singleton group (n = 10)
***HERVWE1*** ** score**	1.43±0.33	1.75±0.33†	1.43±0.35	1.44±0.36	1.32±0.40

Data are shown as the means ± standard deviations.

† P<0.01 vs. other four groups.

**Figure 3:**
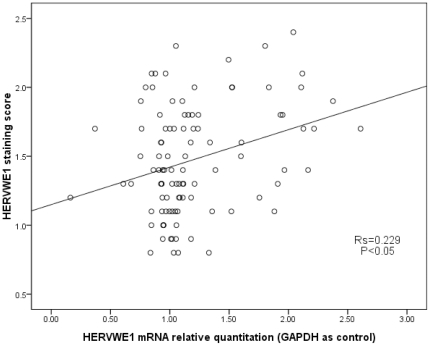
The correlation between the HERVWE1 staining score and the HERVWE1 mRNA level. The scatterplots showed the correlation between the HERVWE1 transcript profile and the HERVWE1 staining score in all five groups. Using GAPDH as internal reference gene, the HERVWE1 transcript level was significant positively correlated with the HERVWE1 staining score (spearman correlation coefficient 0.229, P<0.05).

Based on the Spearman correlation analysis, the *HERVWE1* staining score was positively correlated with the *HERVWE1* mRNA level (R_s_ = 0.229, *P*<0.05, *GAPDH* as control). Figure 3 shows the correlation between the *HERVWE1* staining score and *HERVWE1*mRNA level. The *HERVWE1* protein expression level was consistent with the mRNA expression level in twins. Both of these measurements were increased in the smaller twin in discordant group.

### The methylation profile *of HERVWE1* transcriptional regulation region

**Figure 4:**
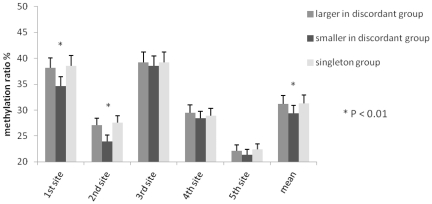
The methylation profile of the HERVWE1 promoter region, as determined by the pyrosequencing assay. There were 5 CG sites in HERVWE1 promoter region. The pyrosequencing assay was applied to investigate the five CG sites methylation profile. The bar chart showed the five CG sites methylation level. All of the CG sites methylation ratios were lower than 40%. The most hypermethylated site was the 3rd CG site. The most hypomethylated site was the 5th CG site. The black, darkgrey, and lightgrey bars were represented the smaller discordant twin group, larger discordant twin group, and singleton group respectively. The 1st, 2nd, and mean methlation level in smaller discordant twin group were lower than that in larger discordant twin group (P<0.01) and singleton group (P<0.01).

Pyrosequencing assay was applied in 21 pairs of discordant twins and 10 singletons. The methylation levels of the 1^st^ and 2^nd^ CG sites and the average methylation levels of the five CG sites were lower in smaller discordant twin group compared with the larger discordant twin group (*P*<0.01), and the singleton group(*P*<0.01). However, no differences were detected at the 3^rd^, 4^th^ and 5^th^ CG sites (*P*>0.05), as summarized in Figure 4. When the CG sites were sorted by methylation level, the order was 3^rd^, 1^st^, 4^th^, 2^nd^ and 5^th^ for both discordant twin groups.

**Figure 5:**
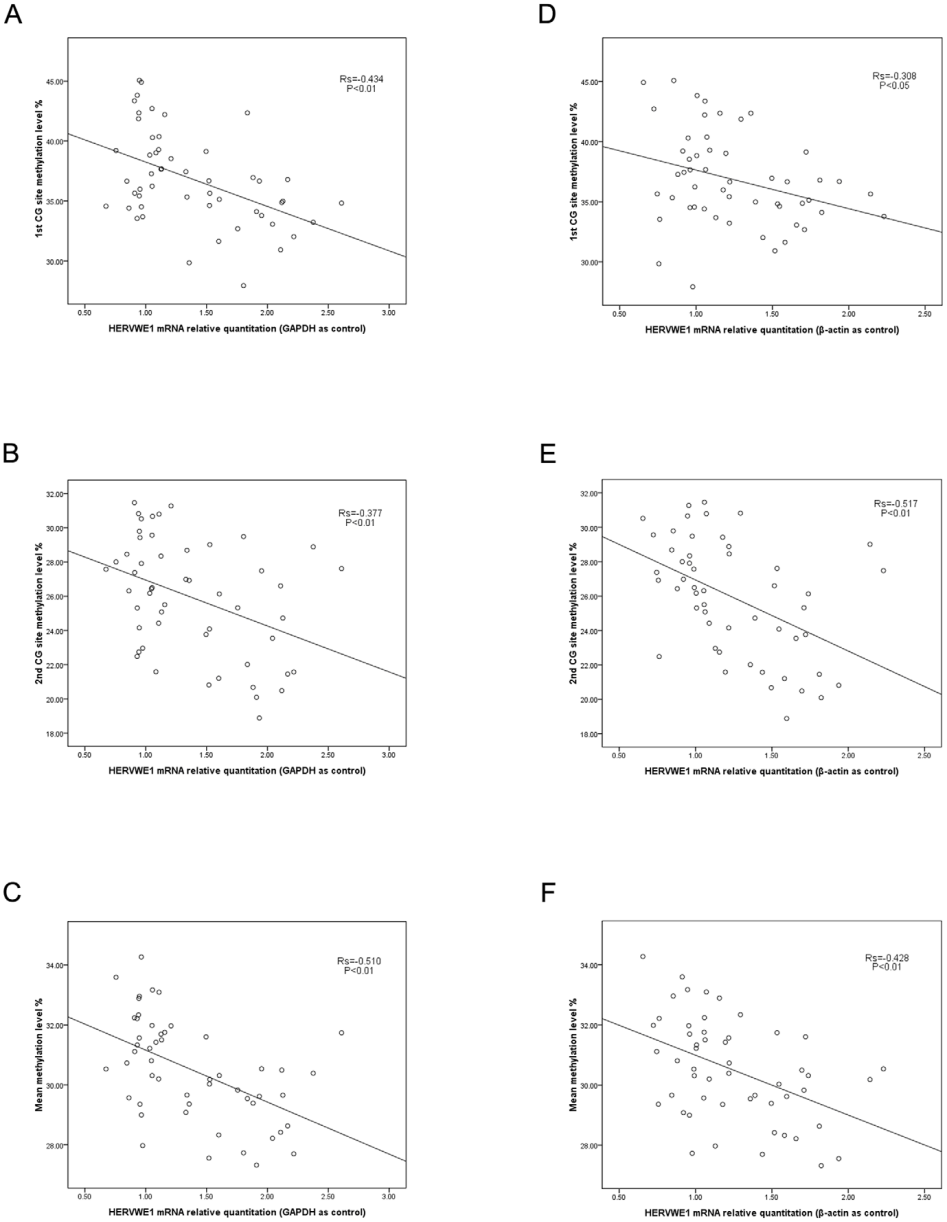
The correlation between HERVWE1 promoter region methylation level and HERVWE1 transcript levels. The scatterplots showed the correlation between the HERVWE1 transcript profile and the HERVWE1 promoter region methylation level in discordant twin groups and singleton group. Using GAPDH as internal reference gene, the HERVWE1 transcript level was significant negatively correlated with the HERVWE1 promoter region methylation level (A–C). Using β-actin as internal reference gene, the HERVWE1 transcript level was significant negatively correlated with the HERVWE1 promoter region methylation level (D–F).

Spearman correlation analysis was applied to study the correlation between *HERVWE1* transcript level and methylation profile. While the *GAPDH* as internal reference gene, the *HERVWE1* transcript level was negatively correlated with the *HERVWE1* promoter region the 1^st^(R_s_ = −0.434, P<0.01), 2^nd^(R_s_ = −0.377, P<0.01),and mean(R_s_ = −0.510, P<0.01) methylation levels (Figure 5). While the β-actin as internal reference gene, the *HERVWE1* transcript level was also negatively correlated with the *HERVWE1* promoter region the 1^st^(R_s_ = −0.308, P<0.05), 2^nd^(R_s_ = −0.517, P<0.01),and mean(R_s_ = −0.428, P<0.01) methylation level (Figure 5).

### Global methylation (5-MC staining)

We used a semi-quantitative analysis method to score the slice density. The staining was located in the nuclei of both of the trophoblast and stroma cells. The positive nuclei were brown, and the negative nuclei were blue after counterstaining with hematoxylin. Comparing the 5-MC staining densities of the larger twin in discordant group, the smaller twin in discordant group and the singleton group samples revealed no significant differences in overall score among the three groups (*P*>0.05) (Table 4).

**Table 4:** 5-MC staining scores for the discordant twin and singleton group.

	Larger twin in discordant group (n = 10)	Smaller twin in discordant group (n = 10)	Singleton group (n = 10)
**Overall 5-MC score**	1.7±0.4	1.6±0.4	1.7±0.4
**5-MC in trophoblast cells**	1.7±0.4	1.2±0.3* †	1.6±0.5

Data are shown as the mean scores ± standard deviations.

* P<0.01 vs. larger twin in discordant group;

† P<0.01 vs. singleton group.

**Figure 6:**
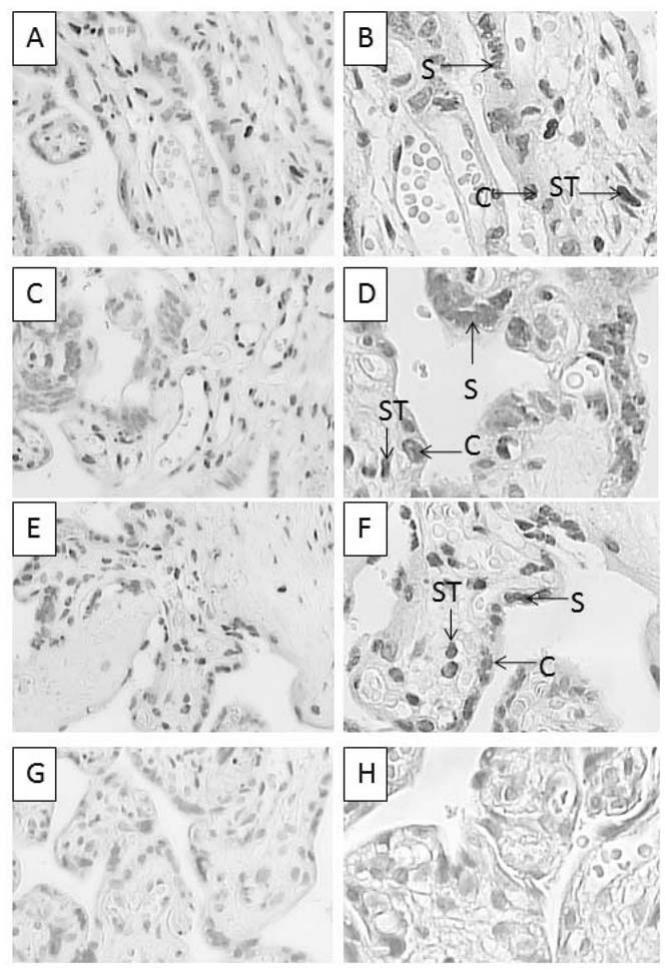
5-MC staining in placentas from discordant twins and singletons. In eukaryotic genomes, DNA methylation always occurs on a cytosine base, the fifth carbon of which is linked to a methyl group (-CH3). IHC staining with an anti-5-MC antibody can quantitatively detect the intra-nuclear 5-MC density and intensity. A–H show placental tissues with anti-5-MC IHC staining. The slices were incubated in a primary sheep polyclonal anti-5-methyl cytosine IgG antibody (diluted 1∶200) overnight. A goat anti-sheep secondary antibody (diluted 1∶400) was applied sequentially. The slices were developed with an immunoperoxidase system. 5-MC was mainly detected in the nucleus. Positive cells were stained brown. Negative cells were stained blue after counterstaining with hematoxylin. (A) The larger infant in discordant group (magnification 10×20). (B) The larger infant in discordant group (magnification 10×40). (C) The smaller infant in discordant group (magnification 10×20). (D) The smaller infant in discordant group (magnification 10×40). (E) A singleton infant (magnification 10×20). (F) A singleton infant (magnification 10×40). (G) The negative control (magnification 10×20). This singleton tissue slice was stained with 1% gelatin instead of the anti-5-MC antibody. (H) The negative control (magnification 10×40). Syncytiotrophoblast and cytotrophoblast cells are marked as S and C respectively. Stromal cells are marked as ST.

Figure 6 shows pictures of individual cases. For the smaller twins in discordant group, we noticed the staining in the trophoblast cells (marked as S or C in Figure 6) was lighter than that seen in the stromal cells (marked as ST). However, the larger twin in discorant group and singleton group had no such distinction. Therefore, we counted only the trophoblast cells using a semi-quantitative analysis method, and we found that the smaller twin in discordant group had a lower 5-MC staining intensity in trophoblast cells than the larger twin in discordant group and the singleton group (*P*<0.01), but no differences between the larger twin in discordant group and the singleton group (*P*>0.05)(Table 4).

### DNMT transcript levels

**Figure 7:**
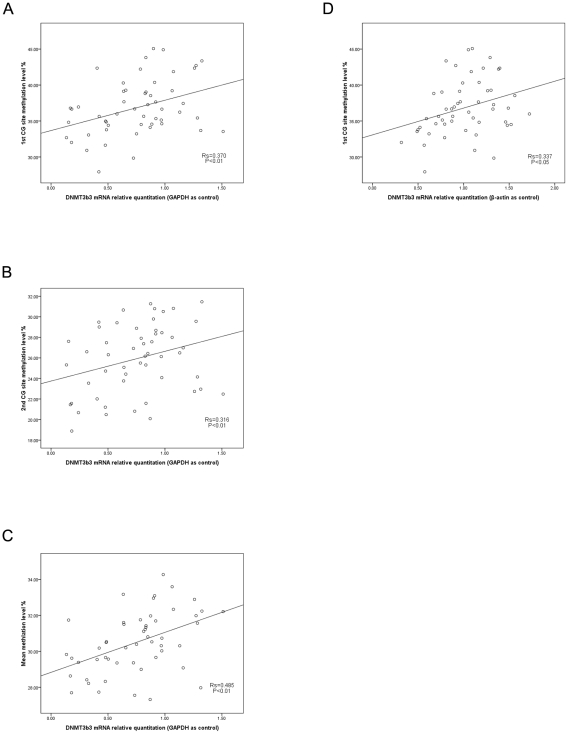
The correlation between DNMT3b3 transcript levels and HERVWE1 promoter region methylation level. The scatterplots showed the correlation between the HERVWE1 promoter region methylation level and the DNMTs transcript profile in discordant twin groups and singleton group. Using GAPDH as internal reference gene, DNMT3b3 transcript level was positively correlated with the HERVWE1 promoter region methylation level (A–C). Using β-actin as internal reference gene, DNMT3b3 transcript level was positively correlated with the HERVWE1 promoter region the 1st CG site methylation level (D).

As indicated above, DNA methylation was closely related with *HERVWE1* transcript level. *DNMTs* are essential for establishing and maintaining the cellular methylation patterns. The alteration of *DNMTs* expression may attribute to the change of methylation pattern. It may be interesting to explore the relationship between the *DNMTs* transcript level and *HERVWE1*methylation profile in the placenta of discordant twins. We used real-time PCR assay to analyze *DNMTs* transcriptional profiles among discordant twin group and singleton group. Thus we calculated the correlations between the *DNMTs* transcript levels and *HERVWE1* methylation profile. While the *GAPDH* as internal reference gene, we only found *DNMT* 3b3 transcript level was positively correlated with the *HERVWE1* promoter region the 1^st^(R_s_ = 0.370, P<0.01), 2^nd^(R_s_ = 0.316, P<0.01),and mean(R_s_ = 0.485, P<0.01) methylation level (Figure 7). While the β-actin as internal reference gene, the result was also true. The *DNMT3b3* transcript level was positively correlated with the *HERVWE1* promoter region the 1^st^(R_s_ = 0.337, P<0.05) methylation level (Figure 7). However, there was no significant correlation between the other *DNMTs* transcript level and the *HERVWE1* promoter region methylation profile.

**Figure 8:**
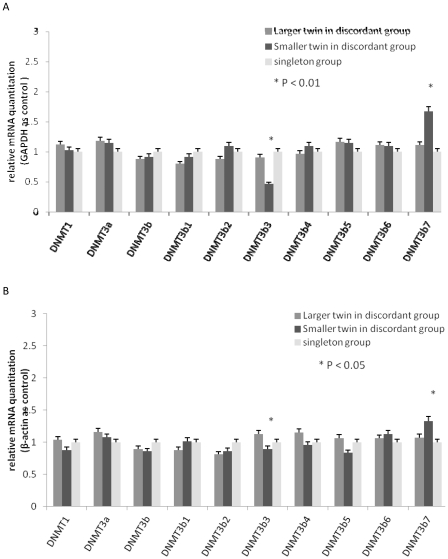
DNMTs transcript levels among discordant twins and singleton group. The values shown indicated the relative quantitation of the DNMT mRNAs, which were standardized to GAPDH (A) and β-actin (B), normalized to the singleton control group. The DNMT 3b3 mRNA decreased and DNMT 3b7 mRNA increased in smaller discordant twin group compared with the larger discordant twin group and singleton group.

We also compared the *DNMTs* transcriptional profiles among discordant twin group and singleton group. The results based on *GAPDH* and β-actin as internal reference gene were consistent. There were no differences between the larger twin and the smaller twin in discordant group for *DNMT1*, *DNMT3a*, or *DNMT3b* (*P*>0.05). The smaller twin in discordant group expressed less *DNMT3b3* (*P*<0.05) and more *DNMT3b7* (*P*<0.05) than the larger twin in discordant group and singleton group. Figure 8 shows *DNMTs* transcriptional profiles based on *GAPDH* and β-actin as internal reference gene.

## Discussion

Our data showed that *HERVWE1* was more highly expressed in smaller twins than larger twins in discordant group. The *HERVWE1* transcript level was negatively correlated with birth weight. The methylation level of *HERVWE1* promoter-region was decreased in the smaller discordant twin group and increased in the larger discordant twin group, also well correlated with *HERVWE1* transcript level. We found that *DNMT3b3* transcript level was positively correlated with the *HERVWE1* promoter region methylation profile. The *DNMT3b3* mRNA level was downregulated, and the *DNMT3b7* mRNA level was upregulated in the smaller discordant twin group.

### *HERVWE1* expression in discordant twins

Because previous studies by Ruebner and coworkers have shown that syncytin-1 is decreased in IUGR, the increase in *HERVWE1* expression in smaller discordant twin placentas is slightly surprising [8], [9]. However, it should be recognized that our study population had unique features; the correlation between these characteristic features and the data has led to some intriguing hypotheses, as discussed below.

Unlike previous studies using singleton placentas conforming to the diagnostic criteria for IUGR, our study subjects were discordant (not IUGR) twins. All of our twins were in the normal range for birth weight based on the twin-specific growth reference curve [26]. In our study, the boundary point for discordancy was 20%, consistent with the majority of the literature. The average weight discordance was 28.5%. Many researchers believe that discordancy itself is not a risk factor for adverse neonatal outcomes [12], [27]. Twin weight discordancy and IUGR have different pathological significance, and they cannot be treated equally.

Both the results from *GAPDH* and *β-actin* as housekeeping gene supported the alteration of *HERVWE1* transcript level in discordant twins. However, after eliminate the contribution of cell proliferation, adjusted by *PCNA*, an indicator of cell proliferation, there was no significant difference between discordant twins. We speculated that the alteration of *HERVWE1* transcript level was related to the cell proliferation, since it has been found that one of *HERVWE1*'s important function is proliferation [3], [28]. Previous study had verified that the *HERVWE1* expression was synchronous with the *PCNA* expression in endometrial carcinoma tissue [28]. This consistent trend between *HERVWE1* and *PCNA* expression in our study further verified the functional effect of *HERVWE1* gene on placenta.

Compensation to hypoxia is one of the characteristics of placenta development. Hypoxia can induce *VEGF* expression and trophoblast invasion in early trimester [29], [30].Hypoxia may also induce ROS production, which is one of features of IUGR and preeclampsia [31], if hypoxia is not corrected. Decreased syncytin-1 expression detected in the placenta of IUGR and the preeclampsia cases seems to be a result of persistent hypoxia. However, increased syncytin-1 expression detected in the smaller discordant twin group may indicate compensatory reactions were involved. The alteration may represent an early stage of IUGR. Increased syncytin-1 may be part of the protective response to hypoxia.

We also found that the larger discordant twin group's *HERVWE1* expression was similar to that of the normal singleton and concordant twin control group, whereas the expression in the smaller discordant twin group was significantly higher than that of the normal singleton group and concordant twin group. If placental function is in the normal range, the placenta of the smaller discordant infant will enlist more compensatory mechanisms, such as release of syncytin-1, to make up for the growth discrepancy. The larger infant's situation is better, and as such, the compensatory mechanism is not as distinct.

The increased *HCS* expression seen in the placentas of the smaller discordant twin group supports our hypothesis. *HCS*, as well as *HCG*, was highly expressed in the syncytium when cytotrophoblast cells merged into syncytiotrophoblast cells. Its expression status indicates placental function [32]. It is well known that *HCS* levels significantly decrease during IUGR and preeclampsia. Attempts have been made by some investigators to use *HCS* or *HCG* as a marker of placental function and an indicator of IUGR severity. The *ß-HCG* protein level was significantly decreased in cytotrophoblast cells fractionated from IUGR placentas in Ruebner's study. In our study, the expression level of *HCS* was consistent with *HERVWE1* expression in each group. Moreover, the HCS transcript levels in both the larger and smaller discordant twin groups were greater than the normal level, as indicated by the singleton and twin control group. This finding indicates that not only did the placental function remain largely intact in both of our discordant twin groups but the alteration of *HERVWE1* expression was also related to the placental compensation capability. Although one of the infants was smaller than the other, the placenta still had the capacity to promote its growth in order for it to catch up to the larger one. This compensatory capability was very different from the decompensated changes seen in the placenta during IUGR.

We applied the analysis of covariance to evaluate the influence of gestational age, since some researchers have found that throughout the entire pregnancy period, *HERVWE1* expression increased as the gestational week increased in singleton pregnancy. In the 3^rd^ trimester, *HERVWE1* mRNA expression reaches its peak, then it drops since 37 wks [33], [34]. This may explain the result of ours were different from Ruebner's study, whose average gestational age of the IUGR group was 36^+3^ wks. We found that gestational age didn't have significant effect on *HERVWE1* transcript expression in each group (P>0.05). We still observed the increased *HERVWE1* transcript level in smaller fetuses of discordant twin when the gestational age was treated as covariate. Furthermore, we couldn't find the correlation between the *HERVWE1* mRNA expression and the gestational age in each group. Thus, the differential expression of *HERVWE1* observed in the study may be an intrinsic alteration for specific mechanisms rather than simple increased gestational age. However, the *HERVWE1* expression trend during the whole twin pregnancy season is still unclear, and we need to enroll more twin cases in future study.

One of our study's merits is that we have found evidence for a differential change in cases of growth discrepancy in twins. This is difficult to study in a singleton case. This novel observation has enriched our understanding of the molecular mechanisms involved in the pathogenesis of fetal growth disorders.

### Methylation alteration in monozygotic twins

Our next important finding is the identification of a particular alteration in methylation in the *HERVWE1* promoter region in discordant, monozygotic twins, with methylation being decreased in the smaller twins. Previous studies in discordant, monozygotic twins also found epigenetic difference between the discordant phenotypes. However, most of these studies were limited to adult disease, such as in schizophrenia and multiple sclerosis [23]. Limited study was conducted in fetal stage. Our study used a unique study population to explore intrauterine growth differences. Only dichorionic twins were enrolled, thereby avoiding the effect of vascular anastomosis between placentas. We found that methylation of the *HERVWE1* promoter region was decreased in the smaller twins but without according changes in global level. It indicated that locus-specific methylation played an important role in regulating gene expression in the placenta during twin development. A meaningful methylation difference definitely existed between the monozygotic twins during the intrauterine stage and have critical influence on fetal or placental development.

### *DNMT* regulation in discordant twins

*DNMTs* may act as key enzymes in a complicated methylation regulation mechanism. In present study, we found that methylation profile of *HERVWE1* transcriptional regulation region was correlated with the alteration of one of the DNMT3b's isoform, *DNMT3b3*, indicating that some factors may control *HERVWE1* expression through the modulation of methyltransferase enzymes. The *DNMT3b* gene has 7 variants, the expression of which depends on different post-transcription spliced patterns [35]. Due to the lack of an intact catalytic domain, both 3b3 and 3b7 have limited catalytic activity. Although some researchers have found that *DNMT3b* variants have diverse expression profiles in cancer tissues [36], the research on these variants and their function is very limited [35], [37], [38]. *DNMT3b3* is a more commonly expressed variant, but its catalytic activity is controversial. We found that *DNMT3b3* transcript levels were lower in the smaller discordant twin group. This result was consistent with the lower methylation levels in the smaller discordant twin group. Without a complete catalytic domain, *DNMT3b7* theoretically has no enzymatic activity. Furthermore, it can competitively bind to the enzyme's binding site on DNA. This mechanism might explain our finding that *DNMT3b7* levels increased in the hypomethylated smaller discordant twin group. This result suggests that *DNMT3b7* acts as a negative regulator of methylation. Overall, the *DNMT3b* variants' tissue expression patterns and functions remain largely unknown. Although our study showed an outline of the expression of *DNMTs* in the placentas of discordant twins, and its potential contributions to methylation regulation in *HERVWE1*, the underneath mechanism is still unclear. Further experiments should focus on normal placental tissue or trophoblast cell lines to explore the relationship between these variants and their functions in fetal growth and placental development.

### Conclusion

We used discordant, monozygotic, dichorionic twins to study *HERVWE1* expression in the placenta and explore its potential impact on fetal growth. We found that *HERVWE1* transcript expression was negatively correlated with infant birth weight in discordant twins. *HERVWE1* expression was increased in smaller discordant twins to levels higher than those seen in normal-growth twins and singletons. CG-site methylation in the *HERVWE1* promoter region maybe a regulatory mechanism for suppressing its expression in the monozygotic placenta. *DNMT3b3* transcript was positively correlated with methylation status in the *HERVWE1* promoter region and its mechanism is worth to be investigated in future study.

## Supporting Information

Appendix Figure S1 **Gene maps of zygosity identification assay.** There are 15 autosomal short tandem repeat loci and 1 gender locus. The name of each locus is marked above the allele. Multiple alleles are possible at each locus. Each wave presents one detectable allele. The serial number of each allele is labeled under each wave. If all of the 16 loci are identical, then the two infants are recognized as monozygotic twins. If any of the genotypes of the 16 loci are different, then the two infants are dizygotic twins. S1A and S1B show two individual infants' genotypes. They were confirmed as monozygotic twins with identical genotypes at each locus.
(TIF)

Appendix Figure S2 **A schematic diagram of the HERVWE1 promoter region CG sites.** The HERVWE1 gene is located at 7q21.2. This is an LTR (long terminal repeat)-element-rich region. Each LTR includes U3 (white), R (dark grey), and U5 (black) regions, in that order. The HERVWE1 transcriptional regulatory element is in the 5′LTR U3 region adjacent to an upstream regulatory element (URE) of composite origin. The URE contains a trophoblast-specific enhancer (TSE, light gray), which confers a high level of expression and placental tropism. TSE and U3 are considered the key methylation and transcriptional regulation control regions. These two regions together are approximately 346 bp in length. There are 7 CG sites in all, two of which are in the TSE; the other five are in the 5′LTR U3. The CAP transcription initiation site is located at the 5′ end of the R region. Taking the CAP transcription initiation site as the zero point, the 7 CG sites are located at −336 bp, −307 bp, −246 bp, −208 bp, −192 bp, −64 bp, and −43 bp. Our methylation study was focused on the first five CG sites. The target CpG dinucleotides are marked with vertical bars and circles.
(TIF)

Appendix Figure S3 **Pyrograms.** Two pyrograms of the same sample. Because pyrosequencing can read through only 50–100 bp for each accurate pyrosequencing primer, we used two pyrosequencing primers to analyze the five CpG sites. The first pyrosequencing primer was used to read the following sequence: TTT TGG GGY GGG TTT TTT TTT TGG GAT GAG GGT AAA AYG TTT GGA GAT ATA GTA ATT ATT TTG. The second pyrosequencing primer was used to read the following sequence: TAG TTG GAT TTT TTA GGT YGA TTA AGA ATT TTT AAG TTT AGT TGG GAA GGT GAT TAY GTT TAT TTT TAA ATA YGG GGT TTG TAA TTT AGT TTA TAT TT. S3A shows the results using the first pyrosequencing primer. The read out sequence was TTTTGGGGYGGGTTTTTTTTTTGGGATGAGGGTAAAAYGTTTGG. This was completely consistent with the targeted sequence. For the first CG site, the C/(C+T) peak ratio was 24%. This result indicates that 24% of CG sites were methylated overall. Similarly, for the second CG site, the methylated cytosine fraction was 3% in total. S3B shows the results using the second pyrosequencing primer. The read out sequence was TAGTTGGATTTTTTAGGTYGATTAAGAATTTTTAAGTTTAGTTGGGAAGGTGATTAYGTTTATTTTTAAATAYGGGGTTT. For the three CG sites, the methylated cytosine levels were 28%, 22% and 18%, respectively.
(TIF)

Appendix Table S1 **Primer Sequences.** The detailed primer sequences for real-time PCR and pyrosequencing assay.
(DOC)
