# Supplementary figures and images for: Increased Expression and Altered Methylation of HERVWE1 in the Human Placentas of Smaller Fetuses from Monozygotic, Dichorionic, Discordant Twins
Source: PLoS One. 2012 Mar 21;7(3):e33503. doi: 10.1371/journal.pone.0033503 (PMC3310130; doi:10.1371/journal.pone.0033503)

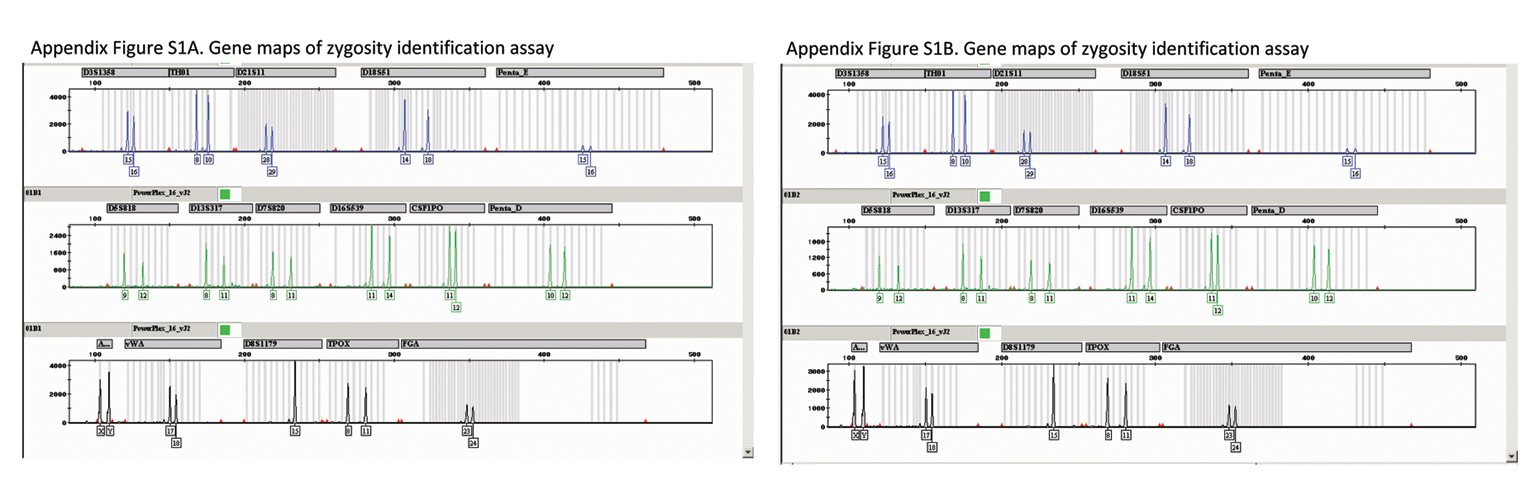

Supplement: Appendix Figure S1 — Gene maps of zygosity identification assay. There are 15 autosomal short tandem repeat loci and 1 gender locus. The name of each locus is marked above the allele. Multiple alleles are possible at each locus. Each wave presents one detectable allele. The serial number of each allele is labeled under each wave. If all of the 16 loci are identical, then the two infants are recognized as monozygotic twins. If any of the genotypes of the 16 loci are different, then the two infants are dizygotic twins. S1A and S1B show two individual infants' genotypes. They were confirmed as monozygotic twins with identical genotypes at each locus. (TIF) [file pone.0033503.s001.tif]

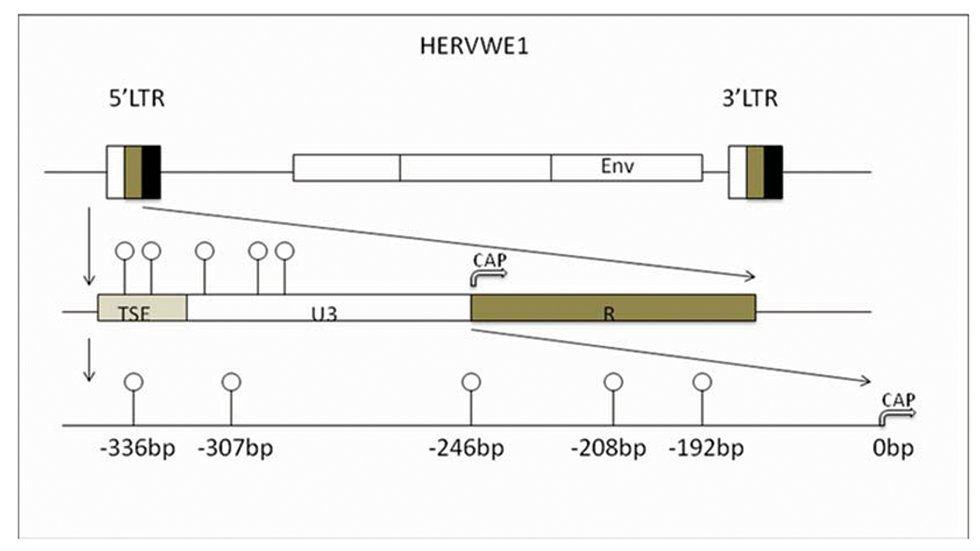

Supplement: Appendix Figure S2 — A schematic diagram of the HERVWE1 promoter region CG sites. The HERVWE1 gene is located at 7q21.2. This is an LTR (long terminal repeat)-element-rich region. Each LTR includes U3 (white), R (dark grey), and U5 (black) regions, in that order. The HERVWE1 transcriptional regulatory element is in the 5′LTR U3 region adjacent to an upstream regulatory element (URE) of composite origin. The URE contains a trophoblast-specific enhancer (TSE, light gray), which confers a high level of expression and placental tropism. TSE and U3 are considered the key methylation and transcriptional regulation control regions. These two regions together are approximately 346 bp in length. There are 7 CG sites in all, two of which are in the TSE; the other five are in the 5′LTR U3. The CAP transcription initiation site is located at the 5′ end of the R region. Taking the CAP transcription initiation site as the zero point, the 7 CG sites are located at −336 bp, −307 bp, −246 bp, −208 bp, −192 bp, −64 bp, and −43 bp. Our methylation study was focused on the first five CG sites. The target CpG dinucleotides are marked with vertical bars and circles. (TIF) [file pone.0033503.s002.tif]

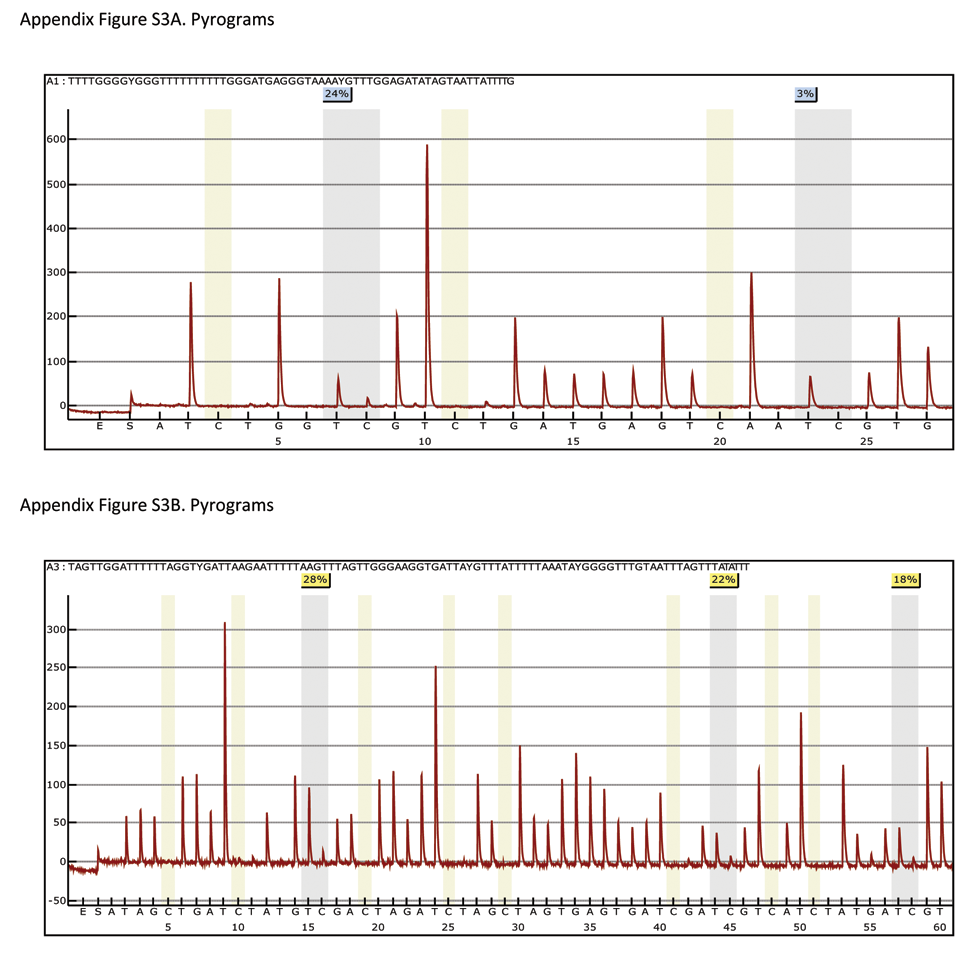

Supplement: Appendix Figure S3 — Pyrograms. Two pyrograms of the same sample. Because pyrosequencing can read through only 50–100 bp for each accurate pyrosequencing primer, we used two pyrosequencing primers to analyze the five CpG sites. The first pyrosequencing primer was used to read the following sequence: TTT TGG GGY GGG TTT TTT TTT TGG GAT GAG GGT AAA AYG TTT GGA GAT ATA GTA ATT ATT TTG. The second pyrosequencing primer was used to read the following sequence: TAG TTG GAT TTT TTA GGT YGA TTA AGA ATT TTT AAG TTT AGT TGG GAA GGT GAT TAY GTT TAT TTT TAA ATA YGG GGT TTG TAA TTT AGT TTA TAT TT. S3A shows the results using the first pyrosequencing primer. The read out sequence was TTTTGGGGYGGGTTTTTTTTTTGGGATGAGGGTAAAAYGTTTGG. This was completely consistent with the targeted sequence. For the first CG site, the C/(C+T) peak ratio was 24%. This result indicates that 24% of CG sites were methylated overall. Similarly, for the second CG site, the methylated cytosine fraction was 3% in total. S3B shows the results using the second pyrosequencing primer. The read out sequence was TAGTTGGATTTTTTAGGTYGATTAAGAATTTTTAAGTTTAGTTGGGAAGGTGATTAYGTTTATTTTTAAATAYGGGGTTT. For the three CG sites, the methylated cytosine levels were 28%, 22% and 18%, respectively. (TIF) [file pone.0033503.s003.tif]
