# Supplementary material for: Increased Expression and Altered Methylation of HERVWE1 in the Human Placentas of Smaller Fetuses from Monozygotic, Dichorionic, Discordant Twins
Source: PLoS One. 2012 Mar 21;7(3):e33503. doi: 10.1371/journal.pone.0033503 (PMC3310130; doi:10.1371/journal.pone.0033503)
Supplement: Appendix Table S1 — Primer Sequences. The detailed primer sequences for real-time PCR and pyrosequencing assay. (DOC) [file pone.0033503.s004.doc]

Appendix Table S1. Primer Sequences

|  | **Gene symbol** | **Gene name** | **Forward primer sequence** | **Reverse primer sequence** | **Product size (bp)** |
| --- | --- | --- | --- | --- | --- |
| **qPCR** | *HERVWE1* | Endogenous retroviral family W, env (C7), member 1 | TCATATCTAAGCCCCGCAAC | CGCCAATGCCAGTACCTAGT | 90 |
|  | DNMT 1 | DNA (cytosine-5) methyltransferase 1 | AGGAGGGCTACCTGGCTAAA | CACTTCCCGGTTGTAAGCAT | 85 |
|  | DNMT 3a | DNA (cytosine-5) methyltransferase 3 alpha | TATTGATGAGCGCACAAGAGAGC | GGGTGTTCCAGGGTAACATTGAG | 111 |
|  | DNMT 3b | DNA (cytosine-5) methyltransferase 3 beta (isoform 1/2/3/6) | GGCAAGTTCTCCGAGGTCTCTG | TGGTACATGGCTTTTCGATAGGA | 113 |
|  | DNMT 3b1 | DNA (cytosine-5) methyltransferase 3 beta isoform 1 | AGAATCAAGGAAATACGAGAACAA | ATCTTCATCCCCTCGGTCTTT | 133 |
|  | DNMT 3b2 | DNA (cytosine-5) methyltransferase 3 beta isoform 2 | GATGAACAGGCCCGTGATAG | TCGAGTTCGACTTGGTGGTT | 119 |
|  | DNMT 3b3 | DNA (cytosine-5) methyltransferase 3 beta isoform 3 | CCGGGATGAACAGGATCTTT | GCACGCTCCAGGACCTTC | 102 |
|  | DNMT 3b4 | DNA (cytosine-5) methyltransferase 3 beta isoform 4 | CGGTTCCTGGAGTGTAATCC | GGTTATTGTCTGTACTTTCTTTAACTGTT | 192 |
|  | DNMT 3b5 | DNA (cytosine-5) methyltransferase 3 beta isoform 5 | AATACAATAGGATAGCCAAGGATCT | TTCAGAGGGGCGAAGAGG | 259 |
|  | DNMT 3b6 | DNA (cytosine-5) methyltransferase 3 beta isoform 6 | CCAAGCTTGGAAAGCATGAA | CCGTTGACGAGGATCGAGT | 89 |
|  | DNMT 3b7 | DNA (cytosine-5) methyltransferase 3 beta isoform 7 | CAGTCTAATTACCTTTCACAGAGAACA | GTCTTGAGGCGCTTGGGT | 102 |
|  | HCS | Placental lactogen (chorionic somatomammotropin hormone) | AGGCTGGAAGACGGCAGCCG | CACAGAGCGGCACTGCACCA | 177 |
|  | GAPDH | Glyceraldehyde-3-phosphate dehydrogenase | AGGTGAAGGTCGGAGTCA | GGTCATTGATGGCAACAA | 99 |
|  | β-actin | Actin, beta | CGCGAGAAGATGACCCAGAT | ACAGCCTGGATAGCAACGTA | 71 |
|  | PCNA | Proliferating cell nuclear antigen | AGGAAGCTGTTACCATAGAGA | ACAACAAGGGGTACATCTGC | 136 |
| **Pyroseq** | *HERVWE1* | Endogenous retroviral family W, env (C7), member 1 | TGTGATATAGTTTTGGATAGTGAATATAGA | biotin-ACCCAAAAAAAAACTACTATTACC | 368 |
